# Plasma Lipoprotein(a) Levels as Determinants of Arterial Stiffening in Hypertension

**DOI:** 10.3390/biomedicines9111510

**Published:** 2021-10-20

**Authors:** Gabriele Brosolo, Andrea Da Porto, Luca Bulfone, Antonio Vacca, Nicole Bertin, Gianluca Colussi, Alessandro Cavarape, Leonardo A. Sechi, Cristiana Catena

**Affiliations:** Clinica Medica, Department of Medicine, University of Udine, 33100 Udine, Italy; gabriele.brosolo@uniud.it (G.B.); andrea.daporto@uniud.it (A.D.P.); luca.bulfone1@gmail.com (L.B.); antonio.vacca94@gmail.com (A.V.); nicole.bertin@uniud.it (N.B.); gianluca.colussi@uniud.it (G.C.); alessandro.cavarape@uniud.it (A.C.)

**Keywords:** arterial stiffness, augmentation index, hypertension, lipoprotein(a), pulse wave velocity

## Abstract

Previous studies have shown that plasma lipoprotein(a) (Lp(a)) plays an important role in the development of hypertensive organ damage. The aim of the present study was to investigate the relationship of Lp(a) with markers of arterial stiffening in hypertension. In 138 essential hypertensive patients free of diabetes, renal failure and cardiovascular complications, we measured plasma lipids and assessed vascular stiffness through the use of pulse wave analysis and calculation of the brachial augmentation index (AIx), and measured the pulse wave velocity (PWV). Plasma Lp(a) levels were significantly and directly related to both AIx (*r* = 0.490; *p* < 0.001) and PWV (*r* = 0.212; *p* = 0.013). Multiple regression analysis showed that AIx was independently correlated with age, C-reactive protein, and plasma Lp(a) (beta 0.326; *p* < 0.001), while PWV was independently and directly correlated with age, and inversely with HDL, but not with plasma Lp(a). Logistic regression indicated that plasma Lp(a) could predict an AIx value above the median for the distribution (*p* = 0.026). Thus, in a highly selective group of patients with hypertension, plasma Lp(a) levels were significantly and directly related to markers of vascular stiffening. Because of the relevance of vascular stiffening to cardiovascular risk, the reduction of Lp(a) levels might be beneficial for cardiovascular protection in patients with hypertension.

## 1. Introduction

Although the propensity of hypertensive patients to develop vascular damage is related to the direct influence of blood pressure levels, additional factors, including circulating lipids, play a crucial role in the process of vascular deterioration. In fact, dyslipidemia is frequently detected in hypertension and serum levels of specific lipoproteins greatly affect cardiovascular morbidity and mortality in hypertensive populations [1].

Lipoprotein(a) (Lp(a)) is a heterogeneous lipoprotein that incorporates a low-density lipoprotein (LDL) particle and the highly polymorphic apolipoprotein(a) (apo(a)) [2]. Serum Lp(a) levels vary over a broad range and the apo(a) gene is the major gene controlling these levels [3]. A number of retrospective and prospective studies have shown that high serum levels of Lp(a) are an independent risk factor for cardiovascular diseases [4]. In addition, previous studies conducted in large groups of patients with high blood pressure have demonstrated that serum Lp(a) levels predict the presence and severity of hypertensive vascular damage [5]. Although the functions of Lp(a) in physiology are unknown, its pathogenic mechanisms have been widely investigated, showing proatherogenic, proinflammatory, and, due to its structural homology with plasminogen [6], prothrombotic effects [4,7]. These effects of Lp(a), together with its interaction with the hemostatic and the renin-angiotensin system, might make it specifically to vascular damage in subjects with high blood pressure [8].

Arterial stiffening is recognized as a strong predictor of cardiovascular events, both in the general population [9,10] and in hypertensive patients [11,12]. Currently, arterial stiffness can be estimated by noninvasive methods, including measurements of augmentation index (AIx) by pulse wave analysis and carotid-femoral pulse wave velocity (PWV). These markers of arterial stiffness are now widely accepted for the assessment of subclinical vascular damage in hypertension [13]. An increase in arterial stiffness has been associated with major cardiovascular risk factors, including being overweight, abnormal glucose and lipid metabolism, and smoking [14,15,16,17].

Because of the relevance of subclinical vascular damage in hypertension, we sought to investigate the relationships between arterial stiffness, as determined by the AIx and PWV, and serum Lp(a) levels in patients with essential hypertension who were free of diabetes, renal failure, and major cardiovascular complications.

## 2. Materials and Methods

### 2.1. Patients

In a cross-sectional study, we included patients with mild (blood pressure from 140/90 to 159/99 mm Hg) to moderate (blood pressure from 160/100 to 179/109 mm Hg) essential hypertension who were consecutively recruited at our hypertension clinic, to which the patients had direct access. The clinic is a regional referral hub for hypertension, to which patients are referred for the assessment of any type of problem related to blood pressure. The patients seen at our clinic are white, include individuals with all grades of hypertension living in North-East Italy, and are representative of hypertensive patients in this geographical area. Blood pressure was measured with an automated device (Omron M6, OMRON Healthcare Co., Kyoto, Japan) after each subject had been supine for 15 min; an average of three readings was recorded. The diagnosis of hypertension was established in all patients according to current guidelines [13]. The exclusion criteria were: age < 18 or >80 years; body mass index (BMI) > 40 kg/m^2^; diabetes; pregnancy; secondary forms of hypertension; treatments that might interfere with Lp(a) levels or with protective effects on the arterial wall, such as antiplatelet drugs or statins; renal failure with 24-h creatinine clearance (CrCl) of less than 60 mL/min/1.73 m;^2^ history of acute illness (<6 months) or history of previous cardiovascular events (stroke, transitory ischemic attack, ischemic heart disease, revascularization, heart failure, claudicatio). Diabetes was excluded by measuring fasting blood glucose, glycated hemoglobin, as well as the patient’s glycemic response to a standard oral glucose test [15]. Cardiovascular complications were identified for exclusion through the analysis of medical records, physical examination, ECG, echocardiography, and ultrasound examination of the aorta, carotid, and iliac femoral arteries. Additional evaluations included exercise testing, myocardial scintigraphy, and angiography, and were performed when indicated [5]. Patients were defined as smokers if they had smoked for at least 5 years and up to 1 year before the study. Alcohol intake was estimated by a questionnaire and expressed as grams/day [18]. Furthermore, due to the beneficial effects of physical training on vascular function, we estimated the level of physical activity of all the patients by a questionnaire. Those patients who performed at least 3 h of aerobic exercise in a week were defined as physically active. The study was performed in accordance with the principles of the Declaration of Helsinki and received approval from the local Institutional Review Board. Informed consent was obtained from all the patients. 

### 2.2. Analytical Methods

A venous sample was drawn without stasis between 8:00 and 9:00 AM after an overnight fast. Plasma glucose was assayed by the glucose-oxidase method. Total cholesterol and triglycerides were assayed by an automated method (International Laboratory, Milan, Italy). HDL cholesterol was assayed after magnesium chloride-dextran sulphate precipitation of apolipoprotein B containing lipoproteins. LDL cholesterol was calculated by the formula of Friedewald. Renal function was evaluated in all patients by serum creatinine and the determination of creatinine clearance in a 24 h urine collection obtained in duplicate. The Lp(a) was measured on serum samples that had been frozen at −70 °C. All the samples were less than 4 weeks old and were stored for an average time of 3 weeks. The concentrations of Lp(a) were determined using a Macra^®^ Lp(a) Enzyme Linked Immunosorbent Assay (ELISA) kit (Trinity Biotech PLC, Bray, Ireland), a method that is highly correlated with the reference method used by the WHO for the standardization of the Lp(a) assay and is not affected by different apo(a) sizes [19]. The intra- and inter-assay coefficients of variation for the lipoprotein(a) measurements were from 2% to 7% and from 6% to 9%, respectively.

### 2.3. Arterial Stiffness

Arterial stiffness was assessed by calculation of the AIx using the pulse wave analysis and measurement of the PWV. An automated device (AtCor Medical SphygmoCor Version 1.2.0.7, Sydney, Australia) was used for the measurements [20]. The same experienced operator performed all the instrumental examinations. The measurements were performed in the morning, when the patients were in a fasted state, and after they had been supine in a quiet room for at least 15 min.

To determine the AIx, the brachial artery pressure waveform was recorded continuously, and the average of at least two records was used for the analysis. The aortic pressure waveform was used to calculate the AIx as the difference in height between the first and the second systolic peaks, expressed as a percentage of pulse pressure [21]. The intra-observer coefficient of variation of the AIx was 8.4%.

To determine the PWV, the distance between the more easily perceived common carotid pulsation and the sternal notch, and between the sternal notch and the superior edge of the femoral cuff, were measured, and these values were loaded onto the device. The time delay between the upstroke of the carotid and the femoral artery pulse waves was measured and the PWV was calculated and expressed in meters per second [22]. The intra-observer coefficient of variation of the PWV was 3.0%.

### 2.4. Statistical Analysis

The values were expressed as mean ± standard deviation. The categorical variables were expressed as absolute number and percentage. The Student *t*-test for the unpaired groups was used for the comparison of the normally distributed variables. Variables with skewed distribution were log-transformed before analysis. The Pearson’s chi-square test was used to compare the frequency distributions. The relationships between the different variables was examined by linear regression analysis and the correlation was expressed by the correlation coefficient. Multivariate regression analysis was performed to ascertain which variables were independently related to the AIx and PWV, which were considered as continuous variables. In this analysis, the variables were sequentially entered in a stepwise model according to the strength of statistical significance obtained in the univariate analysis. Logistic regression analysis was performed to identify which variables were independent predictors of an AIx above the median for the distribution among those identified in the univariate analysis. A receiver operating characteristic (ROC) curve was constructed to depict the clinical sensitivity and specificity of the Lp(a) values to predict an increased augmentation index. A probability value of less than 5% was considered to indicate statistical significance. All the data analyses were performed using Stata 12.1 (StataCorp. LP, College Station, TX, USA).

## 3. Results

We analyzed cross-sectional data from 138 consecutive patients (age: 51 ± 14 years; 66 men, 72 women). A total of 51 (37%) of the 138 patients had never been treated with anti-hypertensive drugs. The remaining 87 (63%) patients were treated with calcium-channel blockers (33%), angiotensin-converting enzyme inhibitors or angiotensin-receptor blockers (36%), beta-blockers (22%), diuretics (25%), and alpha-blockers (4%). 

**Figure 1:**
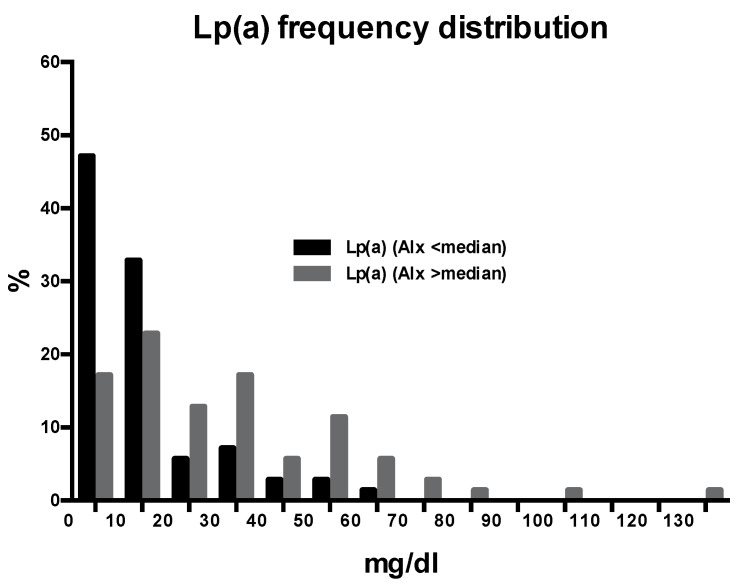
The frequency distribution of plasma Lp(a) levels in hypertensive patients with an augmentation index (AIx) above or below the median value.

The clinical characteristics and biochemical variables of the patients are shown in Table 1, where the patients are also subdivided according to values of AIx below or above the median value (28) of this variable. The patients with an AIx higher than the median were significantly older, more often females and smokers, and had higher total and LDL cholesterol and C-reactive protein than patients with an AIx below the median. Moreover, the AIx was significantly greater in hypertensive patients who were treated with diuretics (32 ± 12 vs. 26 ± 12; *p* = 0.008) or beta-blockers (31 ± 10 vs. 26 ± 12; *p* = 0.045) than in their counterparts, whereas no differences were found between patients treated with other types of antihypertensive drugs. The mean serum Lp(a) levels in the hypertensive patients were 10.4 mg/dl (interquartile range 3.0–31.2). As expected, the distribution was skewed to the left and 36 patients (26%) had levels higher than 30 mg/dl. The frequency distribution of serum Lp(a) levels in patients with AIx below or above the median value is presented in Figure 1, demonstrating that the distribution in the latter group was significantly shifted to higher values (*p* < 0.001). No significant differences in Lp(a) levels were observed between the hypertensive patients treated with antihypertensive drugs and the untreated patients.

The level of PWV was higher in patients who were treated with antihypertensive drugs than in untreated patients (8.1 ± 1.9 vs. 6.9 ± 1.7 m/s, respectively; *p* < 0.001), which was most likely due to a longer duration of hypertensive disease, while no significant differences were observed for gender, smoking habit, alcohol consumption, or physical activity.

**Figure 2:**
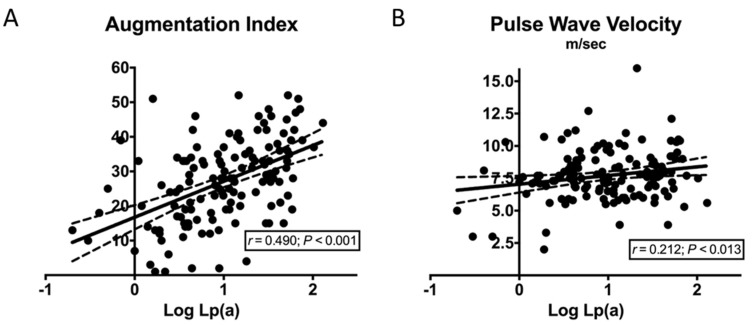
Correlation graphs of Log Lp(a). (**A**) Correlation graph of Log Lp(a) and augmentation index; (**B**) Correlation graph of Log Lp(a) and pulse wave velocity.

According to the univariate correlation analysis, (Table 2), AIx was directly related to age, plasma levels of total and LDL cholesterol, triglycerides, C-reactive protein, and Lp(a) (Figure 2A). PWV was directly related to age, BMI, systolic blood pressure, estimated duration of hypertension, plasma levels of fasting glucose, LDL cholesterol, triglycerides, C-reactive protein, and Lp(a) (Figure 2B), and inversely related to HDL cholesterol.

In the multivariate regression analyses, AIx was independently correlated with age, C-reactive protein, and Lp(a) (beta 0.326; *p* < 0.001) (Table 3). PWV was independently and directly correlated with age, and inversely with HDL, but not with Lp(a) (beta 0.018; *p* = 0.838) (Table 4).

**Figure 3:**
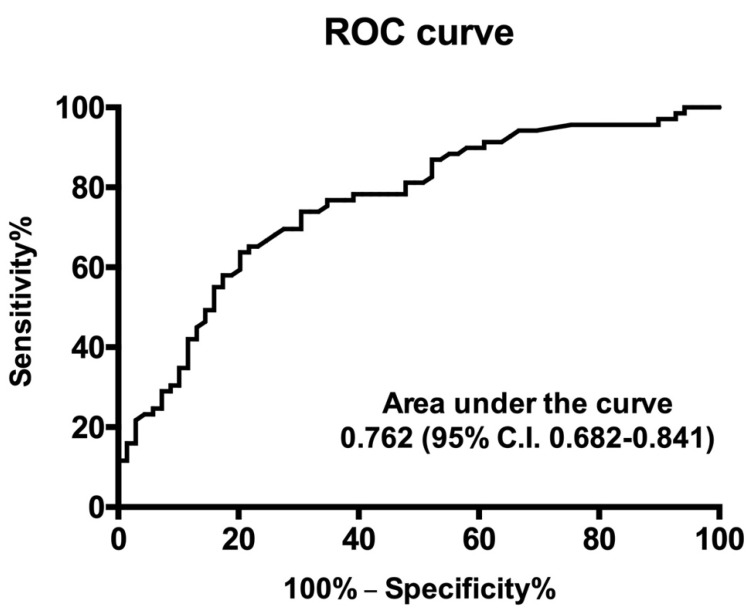
Receiver operating characteristic curve depicting the clinical sensitivity and specificity of plasma Lp(a) values to predict an augmentation index above the median of the distribution. C.I., confidence interval.

Logistic regression analysis was performed to identify independent predictors of AIx values above the median for the distribution, showing that age (odds ratio 1.055, C.I. 1.021–1.097; *p* = 0.005) and plasma Lp(a) (odds ratio 1.026, C.I. 1.003–1.050; *p* = 0.026) were significant predictors. A ROC curve depicting the clinical sensitivity and specificity of Lp(a) values to predict an augmentation index above the median of the distribution is shown in Figure 3.

## 4. Discussion

Many factors, in addition to blood pressure levels, might facilitate the progression of vascular damage in hypertension. In this study, we hypothesized a contribution of Lp(a) to vascular stiffening and tested this hypothesis using noninvasive techniques that reliably estimated the stiffness of the arterial tree. In a large sample of middle-aged, nondiabetic, essential hypertensive patients free of cardiovascular complications and renal failure we found that plasma Lp(a) levels were correlated with the PWV and, independently of possible confounders, with the brachial AIx, thus suggesting a contribution of this lipoprotein to arterial stiffening in hypertension.

Robust epidemiological data support the evidence that high circulating levels of Lp(a) are associated with an increased cardiovascular risk. Lp(a) levels have been independently associated with conditions such as coronary heart disease, stroke, peripheral artery disease, and retinal artery occlusion, and could contribute to cardiovascular risk in subjects with early impairment of renal function and menopausal women [23,24,25,26,27,28]. Significant changes in circulating levels of Lp(a) are associated with ethnicity and dietary changes [4,18,29], and with a variety of conditions that include renal failure, and insulin resistant conditions [30,31]. This is why, in this study, we excluded hypertensive patients with associated diabetes or renal failure.

Progressive arterial stiffening is associated with aging and the process is accelerated by high blood pressure and changes in the levels of circulating lipoproteins that reciprocally interact [32]. Arterial stiffening results from both structural and dynamic changes to the vascular wall [33]. Aging and additional pathological conditions cause extensive anatomical rearrangement with the depletion and fragmentation of elastin fibers and the deposition of collagen elements and matrix metalloproteins, leading to the accumulation of an extracellular matrix. On the other hand, endothelial dysfunction with a reduced release of nitric oxide contributes to arterial stiffening by increasing the resting tone of vascular smooth muscle cells. Additional mechanisms that might have a negative impact on the elastic properties of the arterial tree include: the activation of the tissue-specific renin-angiotensin-aldosterone system, increased production of proinflammatory cytokines with an increase in inflammatory markers, a prothrombotic state, and vitamin D deficiency [34,35,36,37]. Furthermore, heart rate was reported as an independent predictor of PWV in hypertensive patients [38], but we did not observe a significant association between these variables in our patients. Many non-invasive methods have been used for the assessment of arterial stiffness. The measurement of the carotid-femoral PWV is the most popular of these methods, inasmuch as it is inversely related to the elasticity of large conduit vessels. It is generally considered the gold standard. Other methods are based on analysis of the blood pressure waveform, and the AIx is a measure of the wave reflection intensity that is directly related to the stiffness of the entire arterial tree (elastic plus muscular arteries and arterioles) [39]. Therefore, the assessment of PWV and waveform analysis with the calculation of the AIx could be considered a complementary assessment for arterial stiffness. 

In the past, the hypothesis of a possible contribution of Lp(a) to arterial stiffening was investigated in elderly Japanese subjects with type 2 diabetes, and an independent association of its levels with the PWV was reported [40]. However, observations in patients with high blood pressure are scarce [41]. Morishita et al. reported a significant correlation between the plasma levels of oxidized Lp(a) and PWV in a subset of hypertensive patients who were included in a study on coronary artery disease and diabetes [42]. In this study, however, no correction for these important confounders was performed. Similarly, in a study of 34 hypertensive women, Kotani et al. reported a significant relationship between oxidized Lp(a) levels and the cardio-ankle vascular index [43]. Notably, the mean age of the patients in Kotani’s study was 67, and in Morishita’s study it was 66, while the mean age of our patients was 51; the tremendous impact of age on arterial stiffening is well known. We also know how important it is to determine factors predisposing to arterial stiffening at early ages in subjects who are at risk, such as those with high blood pressure. In our study, we measured native Lp(a) with a method that is not influenced by apo(a) size and focused on the pulse wave analysis with calculation of the Aix, together with the PWV. Both vascular variables were directly correlated with higher plasma Lp(a), but the relationship was independent of confounders only for the brachial AIx. One possible explanation could be that some covariates included in the analysis, such as age, body mass index, duration of hypertension, and HDL cholesterol, which had a stronger relationship with PWV on univariate analysis, displayed a greater interaction with this variable than AIx when included in the multivariate model. It is also important to consider that the assessment of arterial stiffness may vary depending upon the site of measurement, inasmuch as the elastic content of the arterial wall decreases progressively from the aortic root to the peripheral arteries, where additional factors might affect vascular distensibility [44]. Because of the relevance of AIx to the assessment of the peripheral arterial tree, the present findings might suggest a greater and more specific relevance of Lp(a) to the peripheral component of arterial stiffness. We also observed that AIx was significantly worse in hypertensive patients who were treated with diuretics or beta-blockers. This observation could be related to the presence of a relatively high hematocrit value, which might have affected blood viscosity for diuretics, and to the vascoconstrictive effects on the peripheral arteries of beta-blockers.

Possible mechanisms linking Lp(a) to arterial stiffening are only hypothetical. Because of the structural homology of Lp(a) with plasminogen, fibrin deposits at the level of the vascular wall could be increased with subsequent activation of transforming-growth factor-α and the stimulation of smooth muscle cell proliferation [45]. Furthermore, increased expression of adhesion molecules, such as E selectin, by endothelial cells [45] might play a role in arterial stiffening together with the proinflammatory actions that have been demonstrated for Lp(a), including the increased production of interleukin-6 by monocytes [46] and of interleukin-8 by macrophages [47].

The major strength of this study is its size, its inclusion of a highly selected group of relatively young patients with high blood pressure, and its use of two different methods to assess arterial stiffness. Some important limitations, however, need to be highlighted. First, the cross-sectional design did not permit us to conclusively establish causality between plasma Lp(a) and arterial stiffening, although the strength and the independence of the association with AIx would suggest so. Second, the use of antihypertensive drugs by a substantial proportion of hypertensive patients might have affected the results, although none of these drugs has been shown to affect Lp(a) levels. Third, a stronger relationship with Lp(a) was observed for the AIx that was obtained at the level of the brachial artery. This might lead to the conclusion that vascular stiffening related to Lp(a) is limited to the periphery of the arterial tree, but the relationship of Lp(a) levels with PWV across the entire arterial system makes this possibility unlikely.

## 5. Conclusions

Because of its high prevalence in the general population, hypertension is commonly considered the most important modifiable cardiovascular risk factor. Arterial vessels are one of the main targets of hypertension-induced damage and a broad interest has gathered around the mechanisms that might contribute to arterial stiffening. Past studies have shown that plasma Lp(a) plays an important role in the development and progression of hypertensive organ damage. This study is the first to demonstrate in a highly selected group of middle-aged patients with uncomplicated hypertension that plasma Lp(a) levels are significantly and directly related to markers of arterial stiffening. This relationship is independent of confounders for the brachial Aix, suggesting preferential effects on the peripheral arterial tree.

These findings have some important clinical implications for the identification of arterial damage in patients with hypertension and for treatment and prognosis. The strength of the relationship between arterial stiffening and Lp(a) implies that this measure might be useful in the diagnostic workup of these patients, to identify those who might be more likely to develop arterial damage. On the other hand, because of the relevance of arterial stiffening as a predictor of future cardiovascular events, a reduction in Lp(a) levels might improve the patient outcomes. As yet, however, no dietary or drug treatments are available to effectively decrease plasma Lp(a) levels, although initial evidence obtained through the use of proprotein convertase subtilisin/kexin type 9 (PCSK9) inhibitors is encouraging. Nonetheless, the detection of elevated Lp(a) levels in hypertensive patients may be useful in improving control over blood pressure and additional risk factors to prevent cardiovascular events. Appropriately designed prospective studies will be needed to test this hypothesis.

## Figures and Tables

**Table 1:** Clinical characteristics and biochemical and instrumental variables of the study patients, subdivided according to the median value of augmentation index (AIx).

	All Patients (*n* = 138)	AIx Below Median (*n* = 75)	AIx Above Median (*n* = 63)	*p*
***Clinical characteristics***				
Age, years	51 ± 14	48 ± 14	55 ± 12	0.002
Males, n (%)	66 (48)	46 (61)	20 (32)	0.001
Body mass index, kg/m^2^	26.8 ± 4.7	26.6 ± 5.2	27.1 ± 4.0	0.563
Heart rate, bpm	68 ± 13	70 ± 12	67 ± 13	0.192
Systolic blood pressure, mmHg	145 ± 18	144 ± 16	146 ± 21	0.435
Diastolic BP, mmHg	89 ± 12	89 ± 10	89 ± 14	0.993
Duration of hypertension, years	8 ± 9	7 ± 9	9 ± 8	0.179
Smokers, n (%)	30 (22)	10 (13)	20 (32)	0.044
Alcohol intake, gr/day	8 ± 12	8 ± 13	8 ± 11	0.924
Physically active, n (%)	36 (26)	22 (29)	14 (22)	0.343
Anti-hypertensive drugs, n (%)	87 (63)	43 (57)	44 (70)	0.129
***Biochemical variables***				
Creatinine cl., ml/min/1.73 m^2^	101 ± 25	99 ± 25	104 ± 25	0.325
Fasting glucose, mg/dl	90 ± 13	90 ± 11	91 ± 14	0.417
Triglycerides, mg/dl	106 ± 56	103 ± 62	111 ± 48	0.397
Total cholesterol, mg/dl	197 ± 45	189 ± 42	205 ± 47	0.040
HDL-cholesterol, mg/dl	57 ± 18	60 ± 19	57 ± 16	0.339
LDL-cholesterol, mg/dl	117 ± 40	110 ± 38	126 ± 40	0.018
C-reactive protein, mg/l	1.16 [0.58–2.10]	0.94 [0.42–1.61]	1.30 [0.76–2.85]	0.003
Lipoprotein(a), mg/dl	10.4 [3.0–31.2]	7.2 [3.0–13.0]	22.4 [8.8–41.2]	<0.001
***Instrumental variables***				
Augmentation index	27 ± 8	18 ± 8	38 ± 7	<0.001
Pulse wave velocity, m/s	7.7 ± 1.9	7.2 ± 1.8	8.3 ± 1.9	<0.001

**Table 2:** Univariate analysis of the relationships between the augmentation index and pulse wave velocity and study variables.

Variables	Augmentation Index			Pulse Wave Velocity
*Clinical characteristics*	*r*	*p*	*r*	*p*
Age	0.311	<0.001	0.501	<0.001
Body mass index	0.101	0.237	0.230	0.007
Heart rate	−0.167	0.055	−0.115	0.187
Systolic blood pressure	−0.018	0.834	0.296	<0.001
Diastolic blood pressure	−0.030	0.795	0.006	0.949
Duration of hypertension	0.043	0.618	0.354	<0.001
Alcohol consumption	0.010	0.912	0.137	0.117
***Biochemical variables***				
Creatinine clearance	0.036	0.677	−0.118	0.170
Fasting glucose	0.064	0.460	0.366	<0.001
Triglycerides	0.175	0.042	0.173	0.044
Total cholesterol	0.268	0.002	0.075	0.387
HDL-cholesterol	−0.135	0.117	−0.334	<0.001
LDL-cholesterol	0.314	<0.001	0.197	0.022
Log C-reactive protein	0.334	<0.001	0.326	0.001
Log Lipoprotein(a)	0.490	<0.001	0.212	0.013

**Table 3:** Multivariate analysis with the augmentation index as the dependent variable.

Variables	β	*p*
Age	0.281	0.002
Gender	−0.078	0.360
Triglycerides	−0.050	0.588
LDL-cholesterol	0.162	0.066
Log C-reactive protein	0.253	0.005
Log Lipoprotein(a)	0.326	<0.001
Diuretics	0.092	0.371
Beta blockers	−0.193	0.072

**Table 4:** Multivariate analysis with the pulse wave velocity as the dependent variable.

Variables	β	*p*
Age	0.328	0.001
Body mass index	−0.034	0.705
Systolic blood pressure	0.104	0.211
Duration of hypertension	0.107	0.283
Antihypertensive therapy	−0.140	0.233
Triglycerides	−0.117	0.197
HDL-cholesterol	−0.331	0.001
LDL-cholesterol	0.139	0.100
Fasting glucose	0.137	0.143
Log C-reactive protein	0.142	0.113
Log Lipoprotein(a)	0.018	0.838

## Data Availability

Not applicable.
